# Overview of psychiatric and behavioral adverse effects of levetiracetam

**DOI:** 10.3389/fneur.2026.1783559

**Published:** 2026-04-16

**Authors:** Amal M. Alkhotani

**Affiliations:** Department of Medicine, Umm Al-Qura University, Makkah, Saudi Arabia

**Keywords:** anti-seizure medications, behavioral adverse effects, epilepsy, levetiracetam, psychiatric complications, psychobehavioral adverse effects

## Abstract

Levetiracetam (LEV) is a broad-spectrum, second-generation anti-seizure medication and is commonly used as a first-line therapy for both generalized and focal epilepsies. A wide range of adverse effects has been reported with LEV, including neurological, dermatological, muscular, and hematological effects. However, psychobehavioral adverse effects (PBAEs) are the most frequently reported. The aim of this review is to explore different psychobehavioural adverse effect of LEV, the risk factors and specific available interventions. Serious but uncommon psychiatric adverse effects include psychosis and suicidality, whereas behavioral adverse effects, such as irritability, aggression, and agitation, are more common. The mean reported rates of irritability, anger, and aggressiveness are 9.9, 2.5, and 2.6%, respectively, with reported discontinuation rates of 2.4–3.4% attributable to aggression and irritability. Several risk factors have been associated with an increased likelihood of developing PBAEs, including a prior history of psychiatric illness, male sex, age <60 years, poorly controlled epilepsy, secondary generalized epilepsy, and absence seizures. In pediatric populations, the reported prevalence of PBAEs ranges from 12 to 38.7%, with a discontinuation rate of approximately 16% due to these adverse effects. Early recognition of PBAEs is essential when initiating LEV therapy. Patients who develop significant PBAE may require dose reduction or discontinuation of LEV, depending on symptom severity. In severe cases, temporary treatment with antipsychotic medications may be required to control behavioral or psychotic symptoms.

## Introduction

Levetiracetam (LEV) is a second-generation anti-seizure medication (ASM). Its anti-seizure effect is mediated through inhibition of excitatory neurotransmitter release via binding to synaptic vesicle protein 2A receptors, which modulates glutamate vesicle exocytosis (1, 2). LEV was initially approved in 1999 by the US Food and Drug Administration (FDA) (3). LEV is a broad-spectrum ASM approved for the treatment of both focal and generalized epilepsies (4). Owing to its broad spectrum efficacy, favorable tolerability, and good safety profile, LEV is widely considered a first-line ASM by neurologists (5). In the United States, LEV accounts for approximately 56% of all epilepsy treatment prescriptions (5). Similar trends have been reported in Germany, where the proportion of LEV prescription for epilepsy increased significantly from 18.0% up to 32.4%, *p* = 0.004 from 2008 to 2020 (6). Furthermore, a recent Cochrane review concluded that LEV is an effective and well-tolerated ASM for the treatment of both focal and generalized epilepsies and identified it as an alternative to other first-line ASMs recommended by the National Institute for Health and Care Excellence in the United Kingdom (7).

LEV is available in both oral and parenteral formulations. The availability of an intravenous preparation facilitates its use in neurocritical care and emergency settings. A meta-analysis evaluating the treatment of benzodiazepine-refractory status epilepticus reported that LEV may be more effective than phenytoin and is associated with fewer adverse effects (8).

However, a range of adverse effects associated with LEV has also been reported. A recent analysis of LEV-related adverse effects using the FDA Adverse Event Reporting System (FAERS) database identified several side effects, the majority of which involved nervous system and psychiatric disorders (9). Adverse effects affecting other organ systems have also been described, including dermatologic, muscular, and hematologic complications. Allergic reactions related to LEV, including Stevens–Johnson syndrome, toxic epidermal necrolysis, drug reaction with eosinophilia and systemic symptoms (9) have been reported, as well as cases of angioedema (10). In addition, rare but serious adverse events, such as rhabdomyolysis (9) and pancytopenia (11), have been associated with LEV use. Despite its favorable efficacy and overall safety profile, psychobehavioral adverse effects (PBAEs) remain one of the leading causes of LEV discontinuation.

This review aims to discuss the spectrum of PBAEs associated with LEV and to examine the risk factors and potential therapeutic interventions for managing these effects.

## Psychobehavioral adverse effects associated with LEV

Patients with epilepsy (PWE) frequently experience multiple comorbidities, among which psychiatric disorders are particularly common (12, 13). The reported prevalence of psychiatric comorbidities in PWE ranges from 13.9 to 37.4% (12, 14, 15). Importantly, the presence of psychiatric comorbidities is associated with significantly impaired quality of life, independent of seizure control (15). The association between epilepsy and psychiatric disorders has been recognized since the 19th century (16), with increasing attention devoted to classification and characterization over time. In 2006, the International League Against Epilepsy (ILAE) proposed a structured classification of psychiatric conditions in PWE. This framework categorized psychiatric manifestations into three groups: (1) psychiatric disorders as comorbidities; (2) psychiatric symptoms that represent manifestations of epileptic seizures; and (3) interictal psychiatric conditions that are specific to epilepsy (16). The epilepsy-specific subcategories include cognitive impairment, epilepsy-related psychosis, affective and somatoform disorders, personality disorders, anxiety disorders, and phobic disorders (16). These conditions should be evaluated in the context of electroencephalographic (EEG) findings and exposure to ASMs. The development of psychobehavioral changes in PWE is often challenging to interpret and requires careful assessment to distinguish seizure-related phenomena from medication-related effects. Since the introduction of LEV, a range of PBAEs has been reported. These adverse effects are commonly categorized into psychiatric adverse effects (PAEs) and behavioral adverse effects (BAEs) (17), although substantial overlap exists between the two categories. BAEs are characterized by behavioral disturbances occurring in the absence of a major psychiatric disorder and most commonly include irritability, aggression, and anger.

## Psychatric adverse effects

Psychosis is a clinical syndrome characterized by the presence of one or more of the following features: hallucinations, delusions, disorganized thoughts, disorganized behaviors, or negative symptoms (18). According to the Diagnostic and Statistical Manual of Mental Disorders, Fifth Edition (DSM-5), psychotic disorders comprise seven distinct diagnostic categories, classified based on symptomatology, duration, etiology, and the presence of associated mood disturbances (19). Psychosis is a recognized comorbidity in epilepsy. Chen et al. categorized epilepsy-related psychosis into five groups, consistent with the ILAE proposal on epilepsy-related psychosis (16, 20). These categories include ASM-related psychosis, interictal psychosis, postictal psychosis, psychosis due to comorbid psychiatric conditions, and substance-induced psychosis. ASM-related psychosis is defined as psychotic symptoms lasting longer than 1 day that interfere with daily functioning and occur during ASM treatment or shortly after ASM initiation, withdrawal, or dose modification. In their study, LEV was the most commonly used medication among patients who developed ASM-related psychosis. Of all patients with ASM-related psychosis 57.1% were receiving LEV, either as monotherapy or in combination with other ASMs. Female sex and temporal lobe epilepsy were significantly associated with the development of ASM-related psychosis. Notably, the occurrence of psychosis was not clearly dose-dependent, as some patients developed symptoms at low doses, while others developed psychosis at higher doses. However, a subsequent study reported symptom improvement following dose reduction (21), suggesting a possible dose-related component in certain individuals. Severe psychiatric symptoms have been reported in approximately 7.8% of the patients receiving LEV therapy, with hallucinations being the most common and severe manifestation. A prior history of psychiatric illness has also been identified as a significant risk factor for the development of severe PAEs (21). Genetic susceptibility to LEV-related psychosis has also been explored. In a genomic study involving 1,106 PWE receiving LEV (22), 149 patients developed BAEs, 37 experienced psychotic reactions, and 920 patients without adverse effects served as controls. Genome-wide association studies (GWAS) did not identify significant univariate genetic variants associated with either BAEs or psychosis. However, analysis of polygenic risk scores (PRS) for schizophrenia demonstrated that patients who had LEV-associated psychosis had an increased risk of common schizophrenia variants compared with controls (*p* = 0.0097) (22). No association was found between LEV-related psychosis and rare schizophrenia-associated variants in SLC6A1, SETD1A, or RBM12 (22). This genomic study was limited to individuals of European ancestry, which restricts its generalizability. Further studies involving diverse ethnic populations and larger cohorts are warranted to validate these findings and to improve the identification of patients at increased risk of LEV-associated psychosis.

Suicidality encompasses suicidal ideation, suicidal thoughts, suicidal attempts, or complete suicide (23). PWE have been shown to have a significantly higher risk of suicidality compared with the general population. A meta-analysis examining suicidality in PWE demonstrated that individuals with epilepsy had substantially higher rates of suicidality than those without epilepsy (24). The pooled event rate was 23.2% among PWE, compared to 3.1% in the general population (24). Several risk factors have been associated with an increased risk of suicidality among PWE, including female sex, poor quality of life, unemployment, limited social support, unmarried status, low income, low educational attainment, antidepressant use, uncontrolled seizures, polytherapy ASMs, early-onset epilepsy, and comorbid depression and anxiety disorders (23). The use of LEV has been associated with an increased risk of suicidality in PWE, with a reported odds ratio (ROR) of 7.6 (95% confidence interval [CI] 1.1–53.7) (25). Since the introduction of LEV, several case reports have described the emergence or worsening of suicidality temporally associated with its use (26, 27). A 2007 study reported that 0.7% of patients receiving LEV experienced suicidality in the context of mood disorders (27). As part of severe PAEs, these manifestations have been shown to improve following dose reduction or discontinuation of LEV (21). Further evidence from a pharmacovigilance study using the FAERS database of ASM-related suicidalities demonstrated that LEV was associated with a significantly increased ROR of 1.286 (*p* < 0.05) (28). However, the present study was limited by its retrospective design and reliance on spontaneous adverse event reporting, which precluded adjustment for patient-related factors and psychiatric comorbidities. Given these findings, high risk patients require close clinical monitoring when initiating LEV, careful assessment for the development of PAEs and screening for suicidalities. Early recognition of such complications allows early interventions to prevent serious adverse effects.

## Behavioral adverse effects

BAEs represent the most commonly encountered PBAEs associated with LEV. BAEs encompass aggression, irritability, and anger, and may occur in the absence of overt psychiatric disorders. Nevertheless, there is significant overlap between BAEs and PAEs, as patients with psychosis frequently exhibit behavioral symptoms, such as aggression, irritability, and anger (21).

Aggression is defined as behavior that causes or is intended to cause pain, discomfort, or harm to oneself or others. It may arise as a defensive response to perceived threats, from goal-directed intent, or as an expression of anger (29). Irritability refers to a state of heightened sensitivity to external stimuli that results in negative affective responses and behavioral dysregulation (29).

In a Japanese study of ASM-associated aggression using the Japanese Adverse Drug Event Report database, the crude ROR for aggression associated with LEV monotherapy was 17.14 (95% CI 10.33–26.90; *p* < 0.01). Combination therapy with perampanel showed a significant association with aggression in polytherapy, with a crude ROR of 18.19 (95% CI 8.13–38.37), and an adjusted ROR of 25.90 (95% CI 1.14–59.10; *p* < 0.01) (30). In the present study, age <60 years was the only significant risk factor for the development of aggression in patients receiving LEV. Another study evaluated the reported adverse effects of LEV using the FAERS system. Among 26,182 adverse effect cases, BAEs were commonly reported. Aggression showed an ROR of 13.37 (*χ*^2^ = 26,073.83), anger 12.3 (*χ*^2^ = 3,849.4), irritability 9.86 (*χ*^2^ = 3,929.99), and agitation 6.01 (*χ*^2^ = 1,606.88) (9).

In a study comparing older with newer ASMs with regard to the occurrence of PAEs and BAEs, LEV had the highest rate of PBAE, at 22.1% (*p* < 0.001, OR = 6.87). Compared with other ASMs, it also had the highest rates of intolerability (17.7%), dose reduction (9.4%), and treatment cessation (8.3%). Factors associated with the occurrence of PAEs and BAEs among all the studied ASMs included a history of psychiatric disease, secondary generalized epilepsy, poorly controlled epilepsy, and a history of absence seizures (17).

In a retrospective analysis of PAEs associated with newer ASMs leading to drug discontinuation, a prior history of psychiatric disease was associated with a significantly higher withdrawal rate (50 of 849; 5.9%; *p* = 0.017). Men were more likely to develop aggressiveness than women (11 of 527, 2.1%; *p* = 0.004) (31).

A study using the FAERS database examined BAEs associated with ASMs and LEV in patients older than 12 years. LEV-associated BAEs were related to male sex, younger age, and concomitant drug use. The onset of BAEs occurred within 4.5 days after initiation of LEV in patients aged 12 years or older and within 1.5 days in younger patients (32).

A recent systematic review analyzing BAEs associated with LEV, topiramate, brivaracetam, and prempanel reported the following rates of LEV-related BAEs: irritability with a weighted mean of 9.9% (1.8–26.7%), anger 2.5% (0.7–54%), and aggressiveness 2.6% (1.4–20%) (33). The dose associated with LEV-related adverse effects ranged from 1,000 to 2,123 mg/day. The discontinuation rate related to adverse effects was 3.4% for irritability (0.9–15.4%) and 2.4% for aggression (0.9–4%). The dose range associated with discontinuation of LEV due to BAEs was 1,000–1,920 mg/day. The typical adult dose of LEV ranges from 1,000 to 3,000 mg/day. The development of BAEs in the LEV setting generally occurred at low to medium dose ranges and was not associated with higher doses. This systematic review did not identify specific risk factors associated with the development of BAEs for individual ASMs.

## LEV effect in special groups

LEV is an ASM commonly used in pediatric patients. Several studies have examined the PBAEs of LEV in this population. LEV is generally a well-tolerated ASMS with a good retention rate; however, the reported incidence of PBAEs ranges from 12 to 38.7% (34–39). The rate of LEV discontinuation due to adverse effects is approximately 16% (34, 35, 37).

In a study comparing new ASMs with older agents, LEV was significantly associated with PBAEs (OR = 3.56, *p* < 0.001). LEV also demonstrated higher rates of intolerability related to PBAE (OR 4.20; *p* < 0.001), dose reduction (OR 4.41; *p* < 0.001), and treatment cessation for PBAE (OR 3.48; *p* < 0.001). Factors associated with the development of PBAEs across all ASMs included a prior history of psychiatric disease, drug-resistant epilepsy, and absence and frontal lobe seizures (35). Another study evaluating BAEs associated with ASMs found no significant differences between valproic acid (VPA) and LEV in terms of behavioral effects. In that study, VPA exhibited the highest rate of BAEs after LEV. The factors associated with BAE were younger age and pre-existing hyperactivity or impulsivity before initiation of ASMs (40).

A previous systematic review of PBAEs in children identified a total of 13 studies involving 727 patients. The most frequently reported adverse effects were hostility, nervousness, and aggression. The discontinuation rate was 2% in one randomized controlled trial (41) which was lower than the rates reported in later prospective and retrospective studies (34, 35, 37).

The effects of LEV have also been studied in patients with learning disabilities (LD). A study comparing the tolerability of LEV in adult patients with and without LD found that patients with LD experienced more BAEs (23%) than those without LD (10%) (42). In a larger study of patients with LD receiving LEV, 118 of 517 PWE had LD. PAEs were observed in 12.7% of patients with LD and included affective disorders, aggressive behavior, emotional liability, agitation, anger, and hostility. A history of status epilepticus and prior psychiatric disease was significantly associated with an increased risk of PAEs (43). Autism is a neurodevelopmental disorder characterized by impairments in communication and social interaction and is associated with an increased risk of epilepsy. The effects of LEV in patients with autism were evaluated in a study of 40 adults with autism compared with 135 adults without autism. Aggression was not significantly associated with autism in multivariate analysis (44).

LEV can be administered parentally and is easier to use than phenytoin among older ASMs. It does not require cardiac monitoring and can be administered rapidly, making it one of the most commonly used ASMs in intensive care units (ICUs). In a study of 965 ICU patients receiving LEV therapy, 60% developed agitation, restlessness, or delirium, and 25% of these patients required antipsychotic treatment. The median time to the development of BAEs was 1.3 days following LEV initiation. These findings raise concerns regarding the high risk of LEV-related BAEs in patients with acute brain injury in the ICU setting. Further studies are required to re-examine the rate of BAEs in the ICU setting (45).

## Clinical implication

LEV is a highly effective ASM commonly used to treat both focal and generalized epilepsies. When initiating LEV in patients with high-risk features, including a prior history of psychiatric disease, male sex, age <60 years, poorly controlled epilepsy, secondary generalized epilepsy, and a history of absence seizures, careful monitoring of PBAEs is required. Accordingly, screening for the development of PBAEs should be incorporated into routine follow-up visits. If symptoms emerge, dose reduction may be considered. In severe cases, LEV may be discontinued or replaced with alternative ASMs associated with fewer PBAEs. Some patients may temporarily require treatment with antipsychotics or antidepressants to control severe symptoms.

Pyridoxine is a water-soluble vitamin that acts as a coenzyme for multiple enzymatic reactions in the body. Several studies have examined the effect of pyridoxine supplementation on LEV-related BAEs in children. A retrospective pediatric study demonstrated that pyridoxine supplementation with LEV resulted in 41–66% improvement in BAEs (46, 47). Another retrospective analysis showed that pyridoxine supplementation was associated with a lower rate of drug discontinuation compared with patients who did not receive supplementation (48). Marino et al. conducted a randomized case–controlled trial evaluating pyridoxine supplementation for LEV-related PBAEs in children and found that 92% of patients receiving pyridoxine supplementation did not require LEV dose adjustment or discontinuation. Improvement was observed after 9.06 ± 3.05 days of supplementation (49). In contrast, a double-blind randomized placebo-controlled trial showed improvement in BAEs in both the pyridoxine and placebo groups over an 8-week period, with no significant difference between groups at 8 weeks. Pyridoxine was found to be safe in this study (50). Furthermore, a systematic review suggested that pyridoxine may be beneficial for LEV-related BAEs; however, significant heterogeneity among studies was noted, and further research is required before routine use can be recommended (51).

A study by Dreischmeier et al. examined the effects of pyridoxine supplementation in adults receiving LEV (52). It showed that 45% of patients had improvement in symptoms, while 55% showed no change, suggesting a potential benefit of pyridoxine. However, larger randomized controlled trials are required to confirm these findings (52).

## Conclusion

LEV is a broad-spectrum, second-generation ASM. LEV-related PBAEs are common and represent one of the limitations of its use. Patients with a prior history of psychiatric disease, male sex, younger age, and poorly controlled epilepsy are at higher risk of developing PBAEs. Recognizing PBAEs is important when evaluating PWE who present with psychobehavioral symptoms, as these manifestations may be medication-related. High risk patients may require careful follow up and assessment of PBAEs upon starting LEV therapy. Patients with PBAEs related to LEV may require dose adjustment or discontinuation of therapy in severe cases. Early recognition of PBAEs allows early interventions to prevent serious adverse effect like suicidality and improve patients quality of life.
